# AHT-ChIP-seq: a completely automated robotic protocol for high-throughput chromatin immunoprecipitation

**DOI:** 10.1186/gb-2013-14-11-r124

**Published:** 2013-11-07

**Authors:** Sarah Aldridge, Stephen Watt, Michael A Quail, Tim Rayner, Margus Lukk, Michael F Bimson, Daniel Gaffney, Duncan T Odom

**Affiliations:** 1 University of Cambridge, Cancer Research UK - Cambridge Institute, Li Ka Shing Centre, Robinson Way, Cambridge, CB2 0RE, UK; 2 Wellcome Trust Sanger Institute, Hinxton, Cambridge, CB10 1HH, UK; 3 Agilent Technologies UK Limited, Mead Road, Yarnton, Kidlington, Oxfordshire, OX5 1QU, United Kingdom

## Abstract

ChIP-seq is an established manually-performed method for identifying DNA-protein interactions genome-wide. Here, we describe a protocol for automated high-throughput (AHT) ChIP-seq. To demonstrate the quality of data obtained using AHT-ChIP-seq, we applied it to five proteins in mouse livers using a single 96-well plate, demonstrating an extremely high degree of qualitative and quantitative reproducibility among biological and technical replicates. We estimated the optimum and minimum recommended cell numbers required to perform AHT-ChIP-seq by running an additional plate using HepG2 and MCF7 cells. With this protocol, commercially available robotics can perform four hundred experiments in five days.

## Background

The ability to decipher regulatory information held in the genome and epigenome is essential to understanding how transcription is controlled or perturbed through natural genetic variation and in diseased states. Chromatin immunoprecipitation followed by high throughput sequencing (ChIP-seq) has become a widely used method to identify regulatory DNA sequence directly occupied by transcription factors, basal transcriptional machinery, and specifically covalently modified histones.

Previous large-scale ChIP studies required enormous manual experiments or large consortia. Genome-wide data sets for epigenetic information have demonstrated the role of chromatin organization in genome function in single cell types [1-3]. These chromatin maps display histone modifications that demarcate different regulatory regions of the genome such as promoters and gene bodies, or regulatory states such as active or repressed transcription. By contrast, to achieve the scale required for the ENCODE consortia, performing approximately 2,100 ChIP experiments required contributions from nine laboratories to profile the DNA binding patterns from numerous factors and multiple cell types [4]. Further research into how this epigenetic layer of information correlates with genotype and phenotype will require hundreds to thousands more protein-DNA binding assays in diverse cell types and/or temporal studies during cell development [5-7].

A recent study implemented semi-automated analysis of multiple transcription factors and epigenetic marks in the transcriptional regulatory networks of a single immune cell type during pathogen response [8]. The powerful method developed in that study [9] permitted characterization of 400 different protein-DNA binding interactions genome-wide, and demonstrated the increase in productivity that would be unlocked by developing completely automated protocols to minimize manual intervention and maximize throughput. Indeed, this landmark study would be the first of many, if ChIP experiments could be fully automated.

The scale afforded by full automation of ChIP experiments would enable disease genomics in patient cohorts that could connect genotype with cellular phenotype. For example, single nucleotide polymorphisms and small genetic aberrations found in natural human genetic variation have been shown to effect transcription factor binding and transcription itself in studies using ChIP-seq [10]. The ability to map at high resolution targets within the genome for factors such as estrogen receptor-α, a major driver of cell growth in breast cancer, in individual tumors has demonstrated the link between a DNA binding protein, its effect on gene regulation, and disease outcome [11].

Finally, the ability to map hundreds of protein-DNA contacts genome-wide in a rapid, reproducible, and fully automated manner would revolutionize the scale and power of interspecies comparisons of transcription factor binding and epigenetics [12,13]. Prior studies have been typically restricted to three to five species of protein-DNA data [14], which have been painstakingly collected using manual ChIP experiments; indeed, the standard ChIP-seq protocol performed in our laboratory, detailed in [15], is laborious. Combined with the increasing number of reference genomes and ChIP-validated antibodies, full ChIP automation could reveal the evolution of combinatorial networks of tissue-specific transcription factors, chromatin state, RNA polymerase binding, and RNA transcription across potentially hundreds of mammalian species.

Here, we report a fully automated high-throughput ChIP-seq (AHT-ChIP-seq) robotics protocol that starts with sonicated chromatin and ends at multiplexed Illumina DNA library preparation. We comprehensively compared the robotic ChIP experiments against CEBPA, a tissue-specific transcription factor, in a well-studied mammalian tissue (mouse liver) to manually obtain protein-DNA mapping experiments. We further demonstrate automated profiling of the genome-wide occupancy of an additional tissue-specific transcription factor (HNF4A), trimethylation of H3K4, RAD21 (a cohesin subunit), and the transcriptional co-activator p300 in liver. Finally, we estimate the minimum and optimal cell numbers a typical ChIP experiment on the plate would require by profiling trimethylation of H3K4 in titrated cell numbers of HepG2 and MCF7 cells, both of which are widely used human cancer models.

## Results and discussion

### Scaling-down ChIP to a 96-well automatable format

We first carried out a series of experiments to optimize the volumes of both input chromatin and antibodies, and eliminate chemically noxious steps employed by standard protocols [15]. For development and evaluation purposes, we utilized the well-characterized liver transcription factor CEBPA as a benchmark [14], because our laboratory primarily uses liver as a model system for interspecies comparisons of ChIP experiments.

A traditional manual experiment for isolating DNA bound to the transcription factor CEBPA would use one-third of an adult mouse liver (well in excess of 100 million cells), 10 μg of antibody bound to 100 μl of Protein G magnetic beads, in a 3 ml hybridization volume. Operating a high-throughput method based around mouse liver would increase animal requirements significantly: based on our current laboratory protocol, 32 mouse livers would be needed to perform 96 ChIP experiments.

First, we established that CEBPA ChIP could be performed using one-sixteenth of a mouse liver in a 200 μl lysis buffer volume using quantitative PCR (Figure S1 in Additional file 1). Using this standard, six mouse livers would suffice to perform 96 ChIP experiments. Antibodies represent a significant proportion of the cost when performing a high-throughput experiment. We therefore tested the affect of antibody concentration on the CEBPA ChIP enrichment. We determined that 2.5 μg of CEBPA antibody when combined with one-sixteenth of an adult mouse liver produced adequate ChIP-seq enrichment (Figure S1 in Additional file 1). Although we expect our guideline amounts will be largely adaptable to typical mammalian ChIP experiments, other specialized ChIP experiments may well require additional optimization of antibodies and cell/tissue quantities.

A phenol-chloroform step has been used to isolate DNA specifically in the final stages of the ChIP reaction. Phenol-chloroform is toxic, corrosive, and thus not ideally suited to unventilated liquid handling robots. We substituted this step with an Ampure XP magnetic beads purification protocol (Beckman Coulter Ltd, High Wycombe, UK) without detriment to the amount of DNA isolated, as per [8,9]. This scaled-down and automation-ready method was then programmed to run on the Agilent NGS Workstation software (Agilent Technologies UK Ltd, Wokingham, UK) as a five-step process (see Materials and Methods). One of the key benefits of automating the workflow was the ability to finely and reproducibly control the liquid handling steps with minimized sample loss at every stage. During development, the pipetting steps were adjusted to meet the following criteria:

1. Produce a homogeneous suspension of magnetic beads through mixing

2. Remove all supernatant without disturbing Protein G beads or Ampure beads whilst plate on a magnet

3. Transfer volumes precisely

4. Mix RIPA solution without foaming.

**Figure 1:**
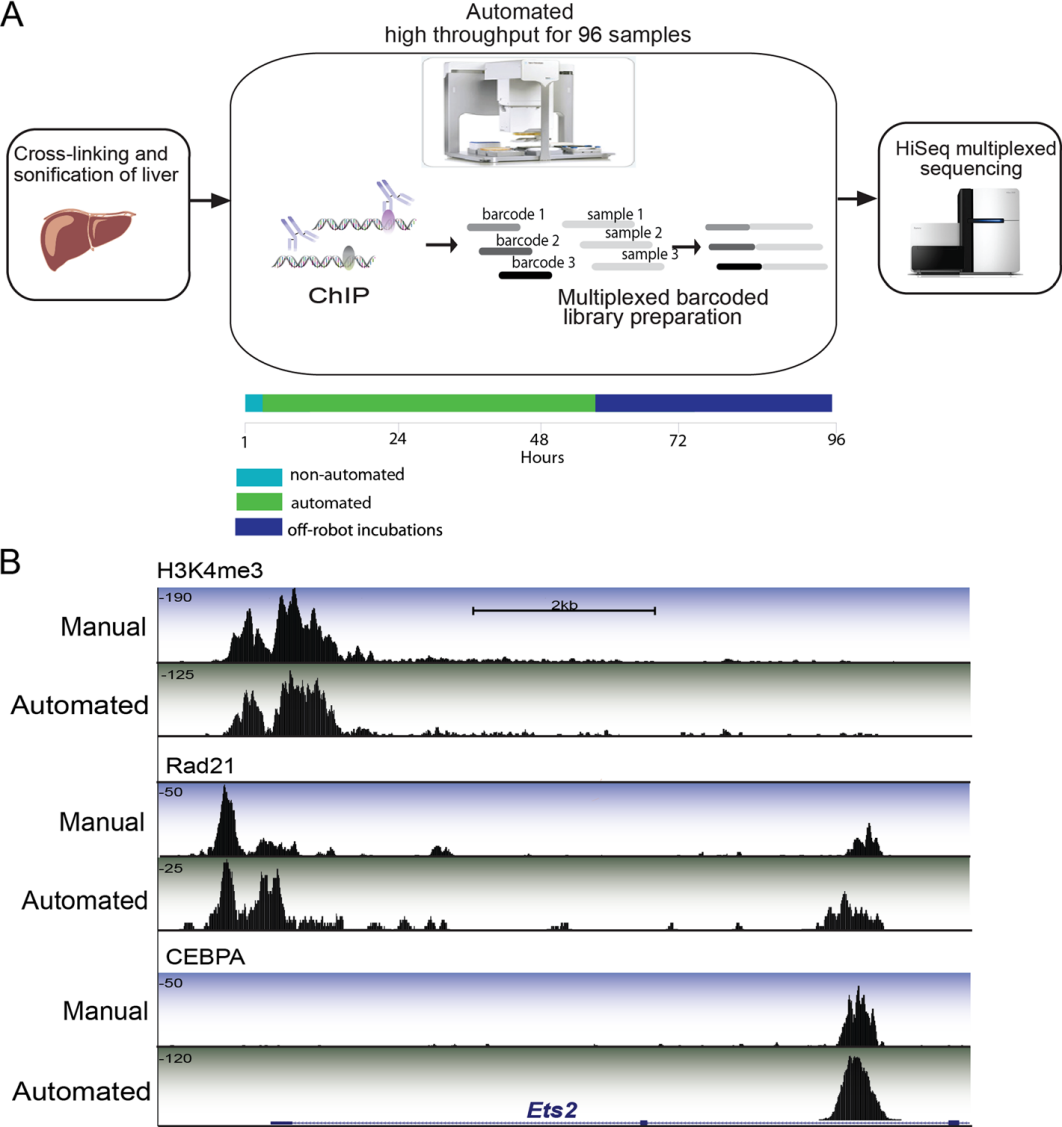
**Schematic of automatic high throughput ChIP-seq protocol and representative data. (A)** Primary tissues were isolated and treated with formaldehyde to crosslink protein-DNA contacts. Sonicated lysate was then transferred to an Agilent workstation where it was added to prepared antibody-bound magnetic beads. Wash steps and purification took place on the Agilent workstation. Illumina library preparation took place on separate automated liquid handling system (Beckman) with subsequent HiSeq2000 sequencing. **(B)** Representation from the UCSC genome browser: a 10 kb region surrounding the *Ets2* gene on mouse chromosome 16. DNA binding regions for three factors are shown: CEBPA, RAD21 and H3K4me3. Tracks with a blue background highlight data acquired from our own previously published manual ChIP-seq experiments. Tracks with green background highlight AHT-ChIP-seq data. The height of each track (y-axis) corresponds with uncorrected read depth. Beneath the enrichment track is the RefSeq genome annotation.

These goals were achieved by adjusting the position of the pipette tip in the well, the velocity and acceleration of the pipette tip into and out of the liquid, and the velocity and acceleration of the syringe plunger. In Additional file 2, we detail each step that was optimized, with the final settings used in the automated protocol. Additional file 3 contains all the files required in order to run the automated protocol on an Agilent Bravo. Automation of the protocol resulted in less user hands-on time and a significant increase in throughput over manual ChIP reactions (Figure 1A and Additional file 1: Figure S2).

### Evaluation of AHT-ChIP-seq and comparison with manual ChIP-seq

To demonstrate the quality and type of data a typical user could obtain from the AHT-ChIP-seq platform, we dissected a set of simultaneously performed ChIP-seq experiments, including both technical and biological replicates, in one representative somatic tissue; together, these experiments capture multiple layers of functional information.

We performed 55 ChIP reactions on a single 96-well plate plus matched sonicated DNA controls. We used five individual mouse livers, each of which was used to generate technical triplicate ChIP experiments for CEBPA and HNF4A; duplicate ChIPs of RAD21 and p300; and singlicate ChIPs of H3K4me3. The ChIP-enriched DNA was then used to create multiplexed libraries for Illumina sequencing reactions (see Materials and Methods), followed by paired-end sequencing on a HiSeq2000 with read length of 75 bp. Reads passing quality control were aligned to the mouse genome (NCBI mm9) using BWA version 0.6.1 [16] (Table S1 in Additional file 4). Aligned data from our own previously published manual data sets [17,18] displayed a high degree of correspondence with AHT-ChIP-seq libraries (Figure 1B) as further detailed below. These three manual experiments were selected as the best examples from over 200 manual CEBPA ChIP-seq experiments available in our laboratory.

First, we directly compared the 15 ChIP-seq replicates of CEBPA obtained using the robot with three CEBPA ChIP-seq experiments our laboratory had previously generated manually (as detailed above). Regions of enrichment for CEBPA-bound DNA were identified in the three manual ChIP-seq experiments and our 15 AHT-ChIP-seq by the peak calling algorithm MACS [19].

As an initial quality control of our ChIP libraries, we performed a cross-correlation analysis to confirm the quality of the ChIP experiments, as has been implemented by the ENCODE consortia [20]. A high-quality ChIP-seq experiment affords a high density of sequencing tags directly surrounding the protein-DNA contact location, which accumulate on both the forward and reverse strands centered around the binding site. By contrast, ChIP-seq experiments with poor ChIP enrichment lack these features, and instead show variable and dispersed distribution of reads across the genome. Further, in poor quality experiments, a phantom peak can be observed when performing cross-correlation analysis, which corresponds with read length. Use of this cross-correlation metric confirmed that 13 out of our 15 CEBPA AHT-ChIP-seq reactions were extremely high quality, and identified two ChIP experiments as lower quality, both from a single mouse (Mmu3), which presented non-specific peaks (Figure S3 in Additional file 1).

**Figure 2:**
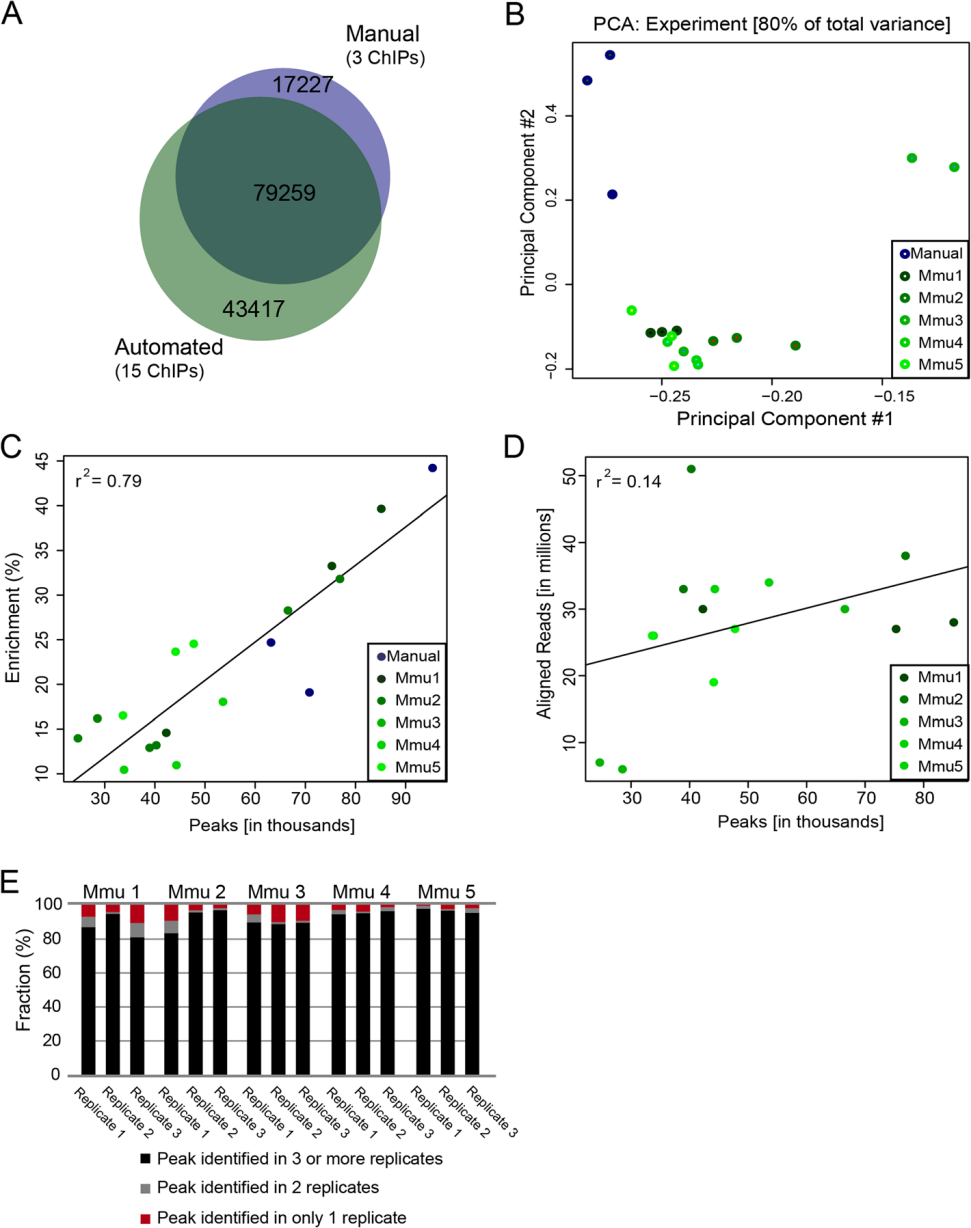
**Comparison of inter-replicate data including our own previously published manual ChIP-seq datasets for CEBPA. (A)** Proportional Venn diagram displaying overlap from the union of peak-sets from published manual data and AHT-ChIP-seq for CEBPA. **(B)** Principal component analysis with automated ChIP-seq data sets (greens) and manual data set (blue). **(C)** Scatter plot of percent enrichment (y-axis) versus number of peaks identified by MACS (x-axis). The Pearson correlation is shown in the upper left corner. AHT-ChIP-seq data sets are indicated in green shades, and manual data sets in blue. **(D)** Scatter plot of aligned reads from the automated ChIP-seq data (y-axis) versus number of peaks identified by MACS (x-axis). Pearson correlation in upper left corner. **(E)** Fraction of unique peaks for each replicate and those that are represented across multiple experiments. Peaks that occur in only one replicate are shown in red, those that occur in at least two replicates in grey, and those which occur in at least three triplicates in black. Mmu1 to Mmu5 refer to biologically individual mice (*Mus musculus*).

We identified the complete, unified set of genomic regions bound by any one or more of the ChIP experiments for both the manual (three replicates) and automated (15 replicates) protocols. We found that the vast majority of CEBPA-bound regions (peaks) identified by the manual protocol were captured by at least one experiment in the automated protocol, and vice versa (Figure 2A): almost 80,000 binding sites were shared.

We next asked what sources of experimental variation contribute to inter-replicate differences in the complete set of CEBPA ChIP experiments. By performing a principal component analysis, utilizing both the genomic intervals (peaks) and aligned reads, we observed a high degree of correspondence between 13 of our AHT-ChIP-seq CEBPA experiments (Figure 2B). The two outliers obtained using AHT-ChIP-seq were the two lower-quality ChIP experiments that showed relatively poor cross-correlation metrics (see above). As might be expected, the three manual ChIP-seq experiments were segregated from the AHT experiments; these experiments were performed at different times by different researchers in tissues from different mouse litters.

Next, we compared how well technical CEBPA ChIP replicates within a single mouse compared to each other. As a reference, we used the three biological replicate experiments performed manually. On average, the AHT-ChIP-seq CEBPA derived experiments identified 27,000 binding sites common to each biological replicate (Figure S4 in Additional file 1). To evaluate the reproducibility of peak calls within our five biological replicates, we used the overlap rate function in the R/Bioconductor DiffBind package [11] (Figure S5 in Additional file 1). In summary, CEBPA-bound intervals were consolidated into union sets for each independent mouse liver; we then asked how many peaks from this union set occurred within increasing numbers of replicates. The degree of overlap among peak-sets for two replicates was about 60% and 40% among three replicates. By this measure, the level of overlap demonstrated that technical reproducibility between matched mouse livers done under exacting conditions was at least equal to experiments carried out using standard manual methods on three separate biological samples.

To further evaluate the effectiveness of our AHT-ChIP-seq method, taking a similar approach to [20], we calculated a simple score for ChIP enrichment as the percentage of aligned reads located within identified peak intervals. We established a benchmark set of genomic intervals bound with high confidence by CEBPA in all three previously published manual ChIP experiments (n = 46,664; median length = 634 bp) (Figure S4 in Additional file 1). We next calculated the proportion of aligned reads that intersected this benchmark set by randomly sampling five million reads from each of our 15 AHT-ChIP-seq and the three manual ChIP-seq experiments, as well as sonicated input DNA control. Using BedTools and controlling for input DNA [21], we identified the fraction of aligned reads located within the benchmark set. There was a high degree of correlation (Pearson r^2^ = 0.79) between the ChIP enrichment score and the number of identified peaks for each replicate (Figure 2C).

The overall quality of robotic reactions was very high, with half the automated replicates comparable to the manual method by these measures, although the ChIP enrichment score for the manual method was modestly higher than the AHT-ChIP-seq method, likely due to the larger amount of tissue and antisera used in manual ChIP reactions. Because there was little correlation between sequencing depth and the number of CEBPA-bound regions identified (Figure 2D), sequencing depth did not appear to limit our ability to capture ChIP-enriched regions. The two exceptions were the lower-quality experiments, (Mmu3_r2 and Mmu3_r3) mentioned above: both were under-represented in their sequencing pools (approximately six million aligned reads) and had the least number of peaks identified by MACS peak calling.

We calculated the fraction of peaks that occurred in one, two, or three-or-more of the 15 AHT replicates (Figure 2E). In every ChIP-seq experiment, the vast majority of CEBPA-bound regions were found in at least two other ChIP experiments; conversely, 5% to 10% were not found in any other ChIP experiment and were thus private to a specific experiment. Collected across all 15 ChIP experiments, privately bound regions numbered in the tens of thousands (Figure 2A).

### Applying motif analysis to confirm CEBPA-bound regions

**Figure 3:**
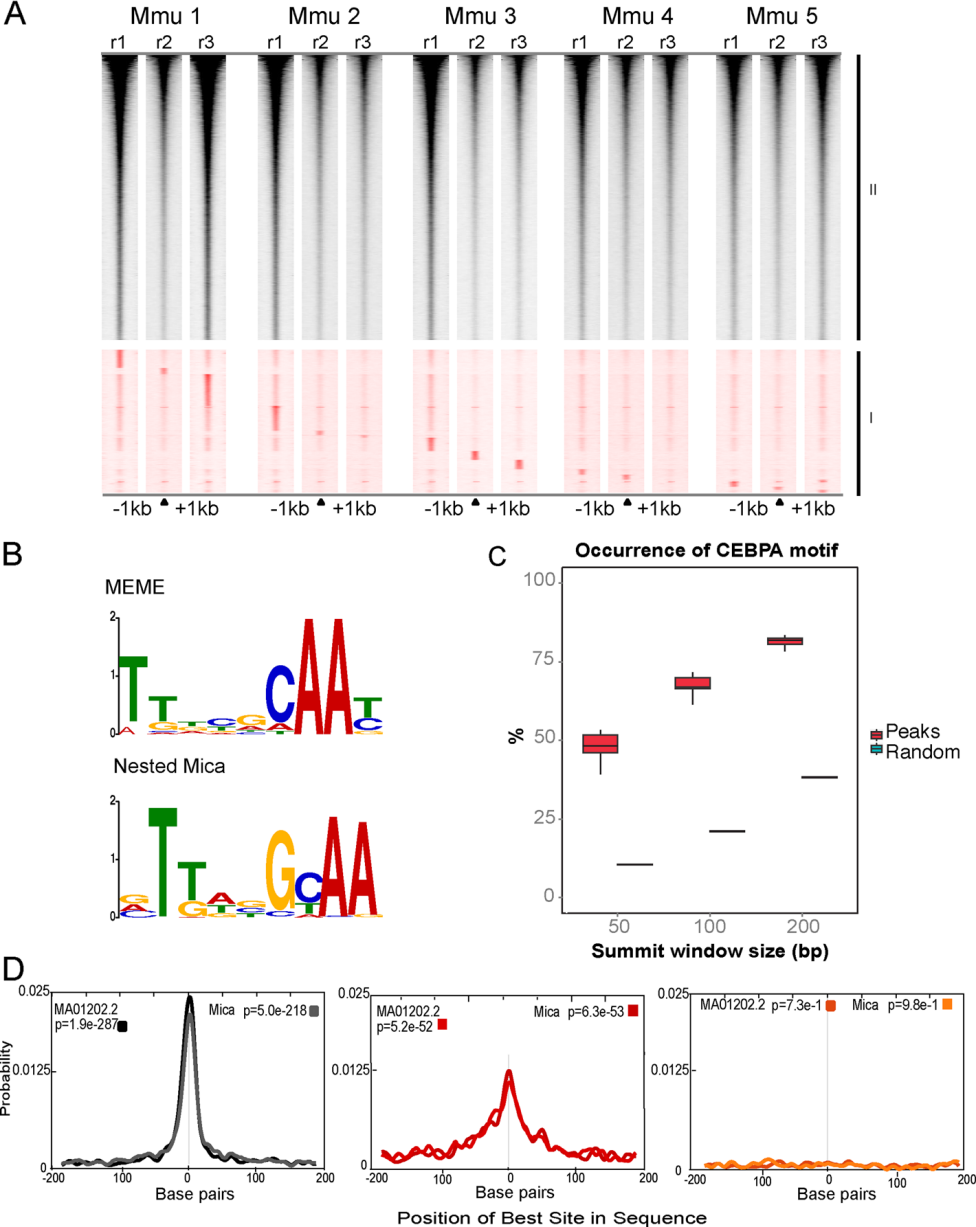
**Many private and low density peaks are real CEBPA binding events. (A)** Heat map of sequence tag density, a one kilobase pair window either side of the center of the peak. The data were then split into two groups, group I (red) contains those peaks that only occurred in one replicate (n = 41,229); Group II (black) contains those peaks that occurred in any two replicates (n = 81,520). Group I peaks are the 15 subsets of replicate unique peaks ordered by Mmu1_r1 unique peaks (top) to Mmu5_r3 (bottom). Group II peaks are ordered by occupancy. **(B)***De novo* motif analyses using MEME and NestedMica software were able to determine a consensus DNA sequence for CEBPA. **(C)** Fractions of peaks (as determined by MACS) containing a CEBPA motif were calculated across all replicates and within all peaks using NestedMica for three summit window sizes: 50, 100 and 200 bp. These were compared with multiple iterations of random sequences using the same summit window sizes. **(D)** For the top 1,000 occupied peaks that occur in multiple replicates (black) and the bottom 1,000 occupied peaks identified in only one replicate (red), both derived consensus motifs were found to be significantly represented in both bins using CentriMo analysis software. A random set of 1,000 genomic intervals of equal size are shown for comparison (orange).

The CEBPA-bound genomic intervals were categorized by how many replicates captured a particular region: Group I regions were identified in one replicate (n = 41,249), and Group II occurred in two or more replicates (n = 81,520). For each of our 15 experiments, sequencing density heat maps were generated for both group I and group II. The group I fraction were ordered by replicate, from Mmu1_r1 top to Mmu5_r3 bottom (Figure 3A). The peaks that were identified as unique to each replicate can be clearly seen with little signal across the other replicates.

All CEBPA peaks, regardless of whether found in group I or group II, had characteristics that reflect direct CEBPA protein binding to a consensus DNA motif. First, the CEBPA motif was present in almost all bound regions. *De novo* motif searches using the MEME-ChIP analysis suite and NestedMica [31,32] identified a CEBPA consensus binding motif in virtually every CEBPA-bound region (Figure 3B). The CEBPA motif was present in 85% of identified peaks with a summit window size of 200 bp, 65% for window size of 100 bp, and 49% for window size of 50 bp. By contrast, similar analyses of random genomic DNA found the CEBPA motif in 35%, 20%, and 12% for summit window sizes of 200 bp, 100 bp, and 50 bp respectively (Figure 3C). Second, in regions identified as bound by CEBPA, the motif was consistently found at the center of the ChIP-enriched region (Figure 3D).

### AHT-ChIP-seq can be used to interrogate protein-DNA binding in cell lines

**Figure 4:**
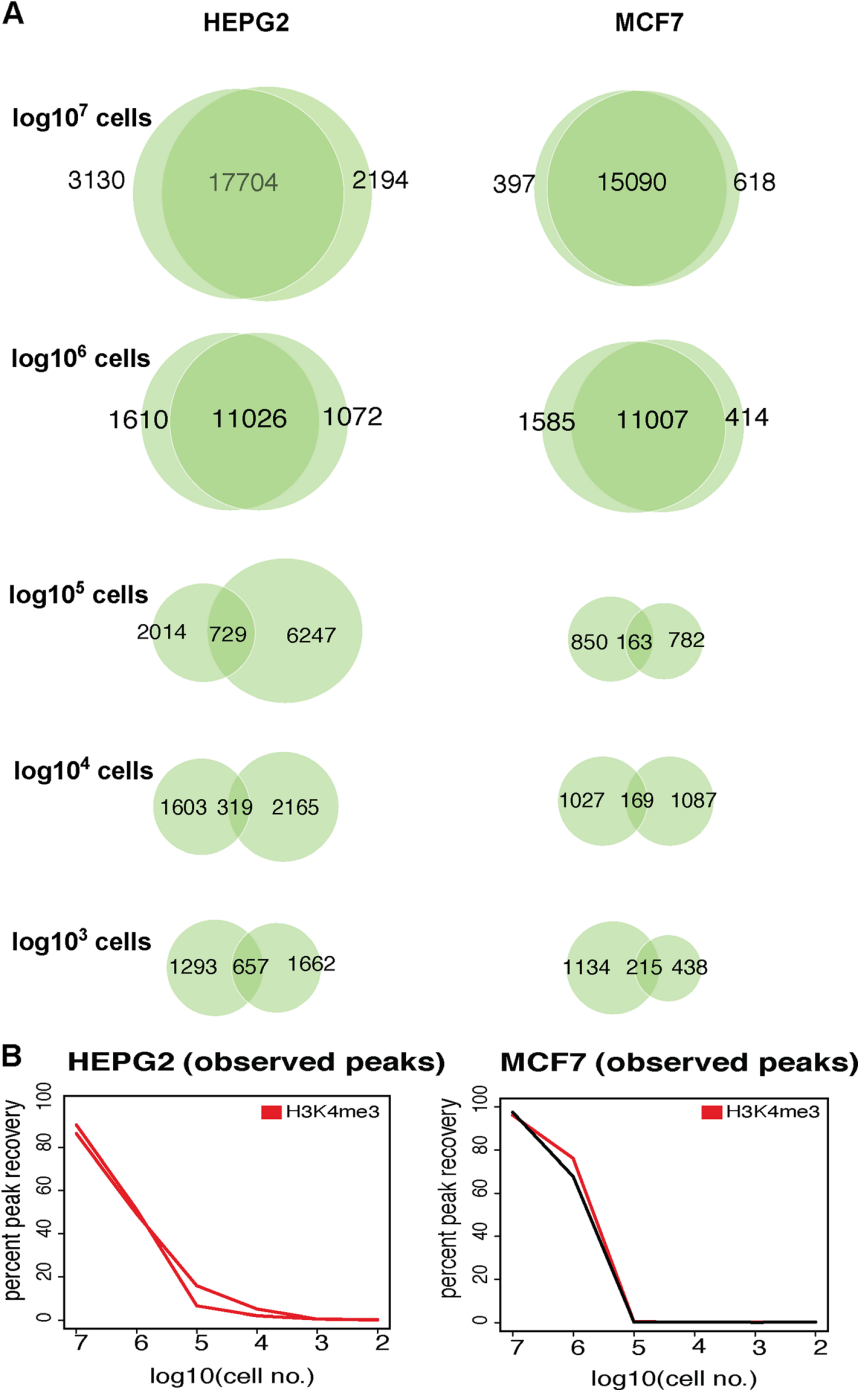
**Titration to ascertain optimum cell number for use in AHT-ChIP-seq experiments. (A)** Proportional Venn diagrams show the overlap of peaks between duplicate experiments for a titration curve of cell number for H3K4me3 in HepG2 and MCF7 cell lines, with highest cell number being log10^7^ and the lowest cell number being log10^3^. No ChIP enrichment was observed for less than 1,000 cells. **(B)** Line graphs showing percentage peak recovery for titration of cell number, with cell number shown as log10 scale with highest cell number being 10 million.

To estimate the number of cells needed for an automated ChIP experiment, we performed H3K4me3 ChIP experiments in two cell lines, HepG2 (hepatocellular carcinoma cell line) and MCF7 (breast cancer cell line), following a titration curve of cell number. For each cell line, we mapped enrichment of trimethylation of H3K4 by performing ChIP in duplicate, using 10 million cells as a maximum down to 100 cells as a minimum, in order-of-magnitude steps. As before, the ChIP-enriched DNA was used to create multiplexed libraries that were sequenced on an Illumina HiSeq2000. Reads passing quality control were aligned to the human genome (NCBI36.3 genome build) using BWA [16] (see Additional file 5) and regions of enrichment for H3K4me3 bound DNA were identified by the peak calling algorithm MACS [19]. We found a high degree of overlap between duplicate experiments when 10 million cells were used (Figure 4A): 17,704 sites for HepG2 and 15,090 sites for MCF7. There was also high degree of overlap between duplicates when one million cells were used, 11,026 for HepG2 and 11,007 for MCF7s, but fewer binding sites overall were found when compared to 10 million cells (Figure 4B). The regions identified as enriched for H3K4me3 were not consistently called by both replicates in cell numbers below one million, but did show some enrichment when manually inspected. Finally, AHT-ChIP-seq failed to yield any H3K4me3 enrichment when only 100 cells were used.

We conclude that for typical human cancer cell lines, 10 million cells is an optimum cell number for use in an AHT-ChIP-seq experiment, and 1 million should be regarded as a minimum number for reproducible ChIP signal (Figure 4A and B).

### AHT-ChIP-seq delivers high quality data on a large scale

**Figure 5:**
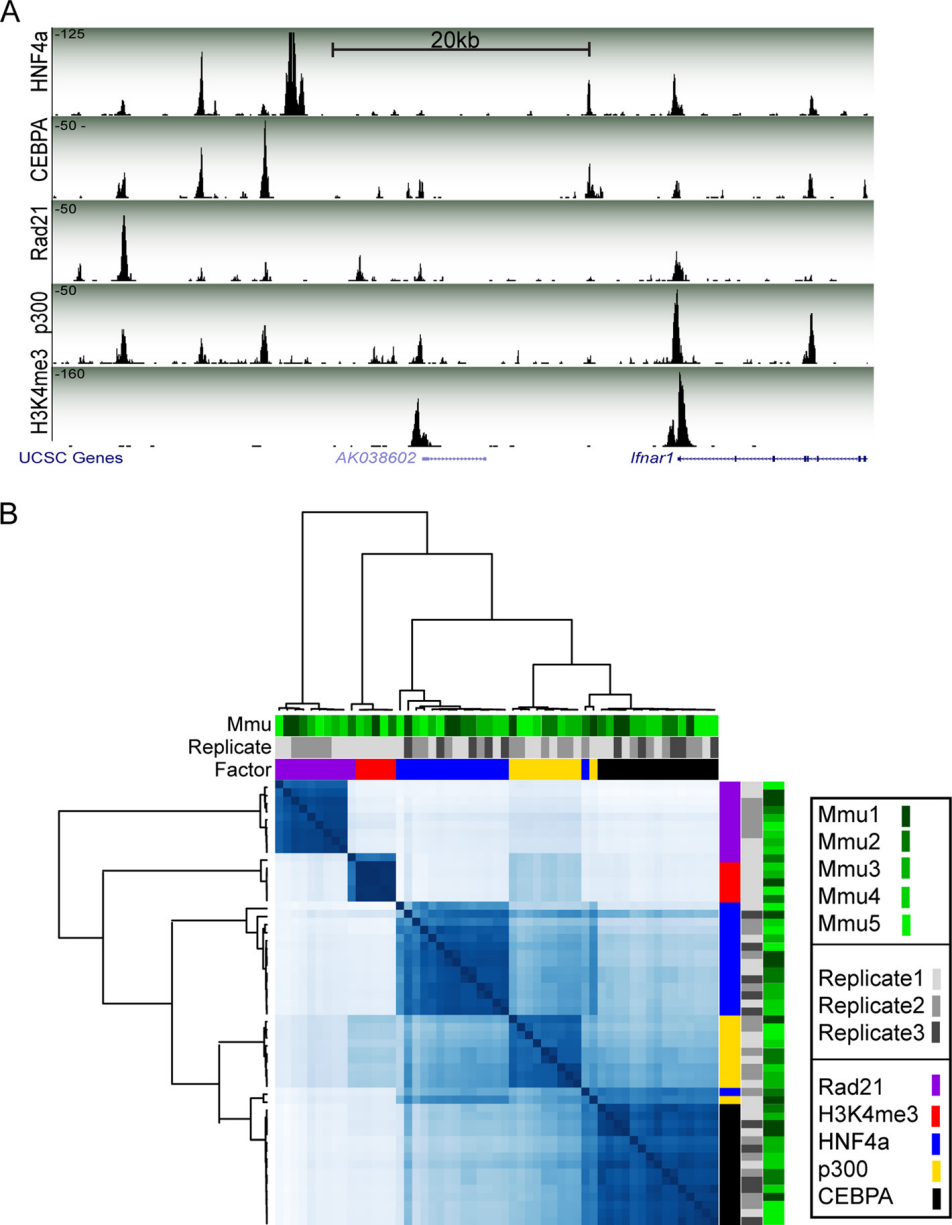
**AHT-ChIP-seq can be used to simultaneously map multiple levels of transcriptional control. (A)** Representation from UCSC genome browser, a 50 kb region around the non-coding RNA AK038602 on mouse chromosome 16. Data generated by AHT-ChIP-seq for five DNA binding regions are shown: HNF4A, CEBPA, RAD21, p300 and H3K4me3. The height of each track (y-axis) corresponds with sequence read depth. Beneath the enrichment track is the RefSeq genome annotation. **(B)** Correlation heat map based on peak location identified by MACS, with three layers of annotation: biological replicate (greens), technical replicate identification (greys) and factors: RAD21 (purple), H3K4me3 (red), HNF4A (blue), p300 (yellow) and CEBPA (black).

Finally, we demonstrated how AHT-ChIP-seq can capture multiple layers of regulatory information in a single experiment by mapping many types of protein-DNA contacts simultaneously in primary mouse liver. We chose factors with diverse regulatory functions and distinct patterns of binding: the DNA binding transcription factor HNF4A [22], which is involved in liver function; RAD21 [23,24], a subunit of cohesin involved in double-strand break repair and chromatid cohesion during mitosis; p300, a non-DNA-binding protein associated with enhancer activity [25,26] (otherwise known as EP300); and H3K4me3, a histone modification associated with transcription start sites of actively transcribed genes [27]. A representative 50 kb region around the non-coding RNA AK038602 is shown in Figure 5A. Peaks were identified as before for all our experiments, and all identified peaks are displayed by correlation heat map (Figure 5B), classified into distinct functional groups. The Mmu2_r1 replicate for RAD21 was highlighted as an outlier by the clustering analysis, and inspection by genome browser showed this ChIP experiment had very low enrichment. With the exception of this single RAD21 ChIP experiment, pair-wise analysis revealed a high level of correspondence between technical replicates for all 20 experiments for p300 and RAD21 (Figures S6 and S7 in Additional file 1).

## Conclusions

To date, most genome-wide characterizations of protein-DNA contacts (such as transcription factor binding and histone marks) have been performed manually in small batches. Recently, liquid handling robotics have been used to partially automate functional genomics protocols based on ChIP experiments [8,9].

Here, we have taken a related approach to create a fully-automated protocol to perform ChIP experiments in a standardized 96-well format after isolation of nuclei and sonication. As the robotics basis for our high-throughput ChIP platform, we selected the Agilent Bravo NGS workstation due to its flexibility and intuitive software. We validated our protocols by comparing multiple (>15) technical and biological ChIP replicates in detail, as well as with published data sets for a representative tissue-specific transcription factor, CEBPA. Our robotics-produced data were of high-quality and in strong concordance with previous manual experiments. Based on list price reagents and excluding cost of final sequencing, it would cost approximately £750 to perform almost 100 ChIP experiments. As the price of next-generation sequencing is dropping and the capabilities for multiplexing several libraries in one lane is increasing, this protocol will be accessible to increasing numbers of laboratories.

Our automation has dramatically increased the typical efficiency of performing ChIP experiments. Manual protocols demand over four hours of hands-on time to perform eight ChIP experiments; by contrast, our robotics protocols require less than two hours of hands-on time to perform 96 ChIP experiments.

The standard protocol we detail permits the simultaneous performance of hundreds of ChIP experiments in parallel with high technical quality. We have demonstrated how rapidly multiple biological replicates of transcription factor binding, chromatin state, and cohesin and co-activator occupancy can be characterized in a primary mammalian tissue. From cell number titration curves for AHT-ChIP-seq, we recommend one million cells as a minimum number for a ChIP experiment, and 10 million as an optimum number.

### Future applications and possible technical developments

#### Reducing cell numbers

Access to adequate patient tissues has been a serious limitation in using functional genomics in the clinic. In part by optimizing the robot’s liquid handling actions throughout the protocol to minimize sample losses, we have modestly reduced the cell numbers needed for a ChIP experiment when performed not only against histone marks, but also CEBPA, a typical tissue-specific transcription factor. Although reducing cell numbers required for ChIP experiments was not our primary focus, the protocol we make available here offers an ideal starting point to optimize clinical genomics experiments to characterize the small amount of tissue found in typical biopsies.

#### Further increasing throughput

The protocols we have developed and report here are functional on any Bravo robot. Agilent offers a 384-well-adapted Bravo robot, which would immediately increase productivity using AHT-ChIP-seq by a factor of four.

#### Reducing hands-on time

The simple addition of a refrigerated reagent carousel would make our protocol entirely hands-free.

## Materials and Methods

### Tissue and cell preparation

ChIP-seq and ChIP-quantitative PCR experiments were performed on liver material isolated from six adult (three-month-old) C57/BL6 male mice obtained from Cancer Research UK Institute and the cell lines HepG2 and MCF7. All tissues were treated post mortem and cells fixed in culture dishes with 1% formaldehyde. The investigation was approved by the CRUK Cambridge Institute ethics committee and followed the CRUK Cambridge Institute guidelines for the use of animals in experimental studies under the UK Home Office license.

### ChIP-seq

ChIP experiments were adapted from those described previously with modifications to allow for scaling to a 200 μl volume for immunoprecipitation, as opposed to laboratory standard 3 ml protocol. Cell lysis and sonication was carried out as previously described [15], with minor modifications as follows: cell lysates were left undiluted and the Triton X-100 volume adjusted appropriately. Protocols carried out on the Agilent Bravo NGS were programmed as a five-step process and the Agilent Vworks Automated ChIP protocol files are available in Additional file 3. All incubation and wash steps were carried out in a Nunc 1.2 ml deep well plate 260251 (Fisher Scientific UK Ltd, Loughborough, UK).

***Step 1: Attachment of antibody to Protein G beads.*** A 25 μl aliquot of Invitrogen Protein G Dynabeads (Life Technologies Ltd, Paisley, UK) was washed with 0.5% BSA/PBS solution followed by addition of 2.5 μg (12.5 μl at 0.2 μg/μl) of antibody. The plate was sealed and transferred to 4°C and mixed for a minimum of four hours on an orbital shaker Grant-bio, PMS1000 (Grant Instruments Ltd, Cambridge, UK). In this experiment, antibodies used were CEBPA sc-9314 (Santa Cruz Biotechnology, Inc. Heidelberg, Germany), HNF4A ARP31946 (Aviva Systems Biology, Corp. San Diego, CA 92121). p300 sc-585 (Santa Cruz Biotechnology Inc.), RAD21 ab992 (Abcam, Cambridge, UK) and H3K4me3 05-1339 (Millipore Ltd, Watford, UK).

***Step 2: Wash and addition of lysate.*** We again washed antibody-bound Protein G beads in 0.5% BSA/PBS solution and added 180 μl of the sonicated lysate to the prepared beads. The plate was returned to a 4°C cold room for overnight mixing and hybridization.

***Step 3: Wash of DNA bound beads.*** ChIP-DNA-bound beads were washed for 10 repetitions in 180 μl cold RIPA solution, transferred to a rigid PCR plate in 50 μl elution buffer and placed in a 65°C thermal cycler, for a minimum of five hours to reverse protein-DNA cross-links.

***Step 4: Removal of beads, RNase and proteinase K treatment.*** We added 50 μl of Tris-EDTA buffer to the beads to dilute SDS in elution buffer. Next, 2 μl RNase AM2269 (Life Technologies) was added to eluted ChIP-DNA and incubated on Bravo deck at 37°C for 30 minutes, followed by 2 μl of proteinase K treatment AM2548 (Life Technologies) at 55°C for one to two hours.

***Step 5: Purification of DNA.*** Phenol and ethanol precipitation was replaced with an Ampure Bead A63881 (Beckman Coulter Ltd, High Wycombe, UK) cleanup step. We added 180 μl of beads (1.8 times volume) to the DNA, followed by two 70% ethanol washes. After the DNA was eluted in 50 μl water or similar elution buffer, it was ready for the Illumina library preparation step.

### Library and sequencing preparation

Illumina sequencing libraries were prepared from ChIP-enriched DNA in 96-well microtiter plates using automated liquid handling robotic platforms. Pre-PCR library preparation steps were carried out using a Beckman Fx^p^ dual arm instrument with a Cytomat Microplate Hotel (Beckman Coulter Ltd) (for method see Figure S8 in Additional file 1 and Additional file 6). Briefly, 50 μl of DNA was purified by binding to twice the volume of AMPure XP beads (Beckman Coulter Ltd) and eluted in 30 μl of 10 mM Tris-HCl, pH 8.5. End-repair, A-tailing and paired-end adapter ligation were performed using NEBnext reagents E6000S (New England Biolabs, Hitchin, UK), with purification using a 1:1 ratio of AMPure XP to sample between each reaction. Illumina paired-end adapters were used at a final concentration of 20 pM (a 1:20 dilution of our standard library adapter concentration) to reduce adapter dimer formation. After ligation, excess adapters and adapter dimers were removed using two Ampure XP cleanups, first with a 0.7:1 ratio of standard Ampure XP to sample, followed by a 1:1 ratio, with elution in 30 μl of 10 mM Tris-HCl, pH 8.5. We then used 10 μl of this adapter ligated material as a template for PCR amplification with Kapa HiFi 2x Mastermix KK2602 (Kapa Biosystems, Inc. Woburn, MA 01801, US) with 200 nM final concentration of standard PE1.0 and modified multiplexing PE2.0 primers (see Table S2 in Additional file 7). After PCR setup on the Beckman Fx^p^, PCR reactions were cycled on an MJ Tetrad thermal cycler with the following conditions: 94°C for 2 minutes; 18 cycles of 94°C for 20 seconds, 65°C for 30 seconds, 72°C for 30 seconds; and 72°C for 3 minutes.

After PCR, excess primers and any primer dimers were removed by performing a 0.7:1 Ampure XP cleanup on a Caliper Zephyr liquid handler (Perkin Elmer, Waltham, MA 02451, USA) with elution in 30 μl of 10 mM Tris-HCl, pH 8.5. Libraries were pooled in equal volume and the concentration of that pool determined by real-time PCR using the SYBR Fast Illumina Library Quantification Kit (Kapa Biosystems, Inc.) before sequencing on an Illumina MiSeq, 50 cycles single end plus index read, to determine the relative representation of each barcoded library. Based on this data the library pool was reblended so as to give equal representation of each library, and requantified by real-time PCR as above, before sequencing on an Illumina HiSeq 2000 for 75 cycles paired end, plus index read.

### Real-time PCR

Evaluation of ChIP enrichment by quantitative real-time PCR was performed using an ABI7900-HT system as per manufacturer’s instructions (Life Technologies). Reactions were performed using Power SYBR Mastermix (Life Technologies). Samples were normalized to a standard curve of sonicated input DNA over negative control regions. Primers used can be found in Table S3 in Additional file 8.

### Read mapping and sequencing data analysis

Reads were aligned to reference genome mouse build (NCBI mm9) or human NCBI36.3 genome build using BWA version 0.6.1 using default parameters. Ambiguous reads that mapped to more than one region in the genome and those with a mapping score of zero were removed. Regions of ChIP enrichment (peaks) were identified using MACS [19] versions 1.3.7.1 and 1.4.2 against a matched input DNA control of similar read depth.

Intersection and pair-wise comparison of data sets was carried out using Galaxy server [28]. Obtaining counts within intervals was performed using BedTools within the Galaxy server [21].

Principal component analysis, peak overlap rates, determining peak occupancy and peak clustering were all carried out using functions of the R/Bioconductor package DiffBind version 1.4.2 [29].

Heat maps were created using the SeqMINER package [30].

*De novo* motif analysis was carried out using the MEME-ChIP analysis suite and NestedMica. To assess significance of enrichment for CEBPA motifs, NestedMica [31,32] was used to detect motifs in whole peaks. Each replicate was compared to a null distribution generated from 1,000 sets of random intervals with the same width distribution. In each case the observed enrichment was highly significant (Wilcoxon test P <0.001). The overall significance of enrichment across all replicates was calculated using a Wilcoxon test comparing the observed motif content to that expected based on the modes of the null distributions. CentriMo [33] was used to calculate the significance of CEBPA motif within a region 200 bp to either side of the peak summit. Peak summits were obtained from the output provided by MACS. Random intervals were obtained using RSAT analysis suite [34].

Proportional Venn diagrams were created using BioVenn [35].

### Accession code

Data sets are available from ArrayExpress under the accession number: E-MTAB-1579, previously published data sets taken from E-MTAB-941 and E-MTAB-1414.

## Abbreviations

AHT-ChIP-seq: automated high-throughput chromatin immunoprecipitation; bp: base pair; BSA: bovine serum albumin; ChIP: chromatin immunoprecipitation; PBS: phosphate-buffered saline; PCR: polymerase chai reaction.

## Competing interests

The authors declare that they have no competing interests.

## Authors’ contributions

SW, SA and DTO conceived and designed the experiments. SW and SA performed chromatin immunoprecipitation experiments. MAQ performed Illumina library preparations for sequencing. SW, SA and MFB designed automated protocol. SW analyzed the data with support from TR and ML. SA, DG and DTO managed the study. SW, SA and DTO wrote the manuscript with critical input from all the authors. All authors read and approved the final manuscript.

## Supplementary Material

Additional file 1 Supplementary Figures 1-8. Click here for file

Additional file 2 Optimized and final settings for Agilent Bravo AHT-ChIP protocol. Click here for file

Additional file 3 Agilent file for automated ChIP method. Click here for file

Additional file 4: Table S1 MetaData, sequencing metrics, peak calls, file information for liver experiments. Click here for file

Additional file 5 MetaData, sequencing metrics, peak calls, file information for cell-line experiments. Click here for file

Additional file 6 **Beckman.** BMF file for Illumina library preparation method. Click here for file

Additional file 7: Table S2 Illumina library oligonucleotide sequences. Click here for file

Additional file 8: Table S3 Primer sequences used in ChIP real-time PCR. Click here for file
