# Supplementary material for: AHT-ChIP-seq: a completely automated robotic protocol for high-throughput chromatin immunoprecipitation
Source: Genome Biol. 2013 Nov 7;14(11):R124. doi: 10.1186/gb-2013-14-11-r124 (PMC4053851; doi:10.1186/gb-2013-14-11-r124)

Supplementary File Y. Screen shot of Biomek software illustrating Fx deck layout

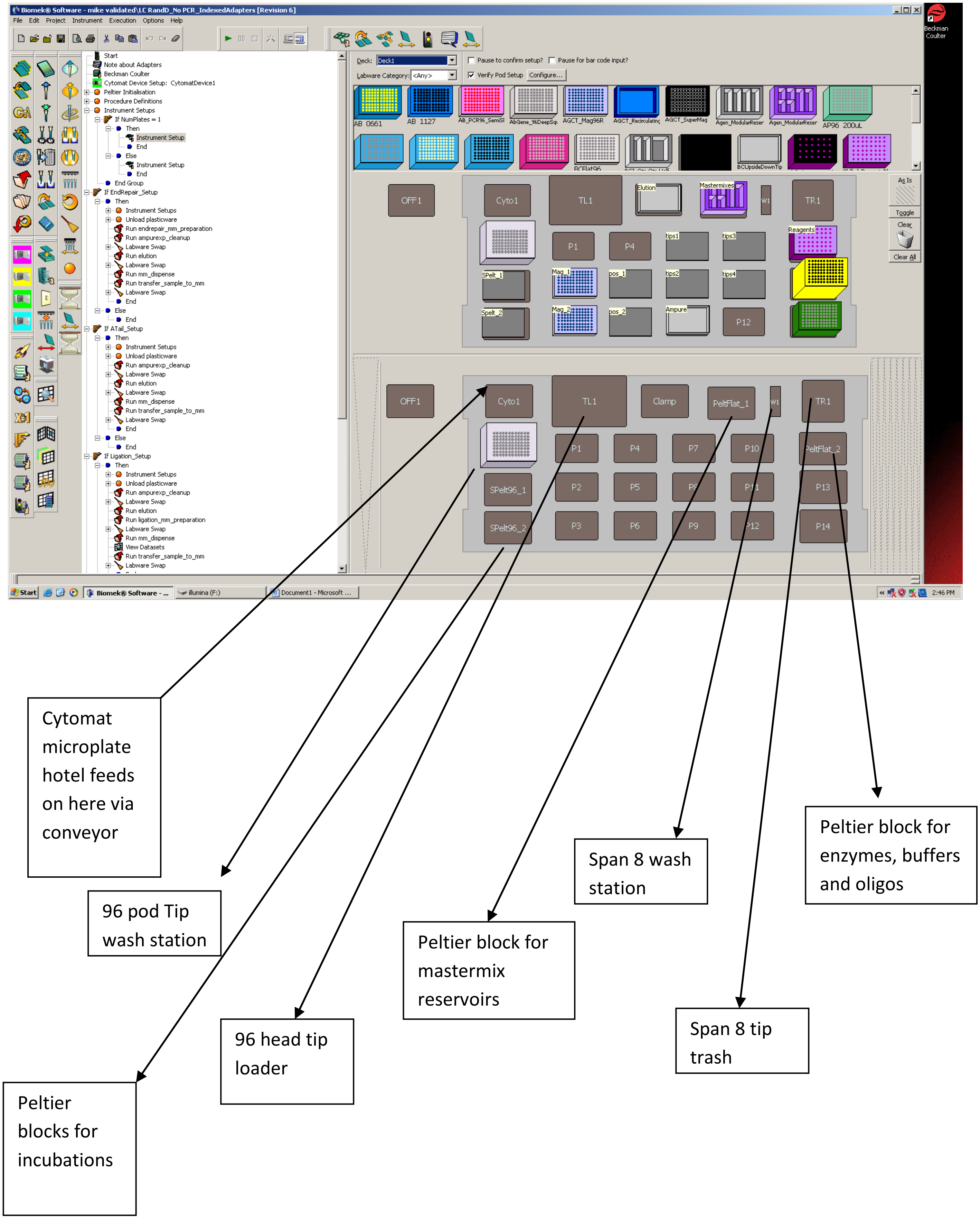

Supplement: Additional file 1 — Supplementary Figures 1-8. [file gb-2013-14-11-r124-S1.zip › Supplementary_figure_8.pdf]
