# Supplementary figures and images for: AHT-ChIP-seq: a completely automated robotic protocol for high-throughput chromatin immunoprecipitation
Source: Genome Biol. 2013 Nov 7;14(11):R124. doi: 10.1186/gb-2013-14-11-r124 (PMC4053851; doi:10.1186/gb-2013-14-11-r124)

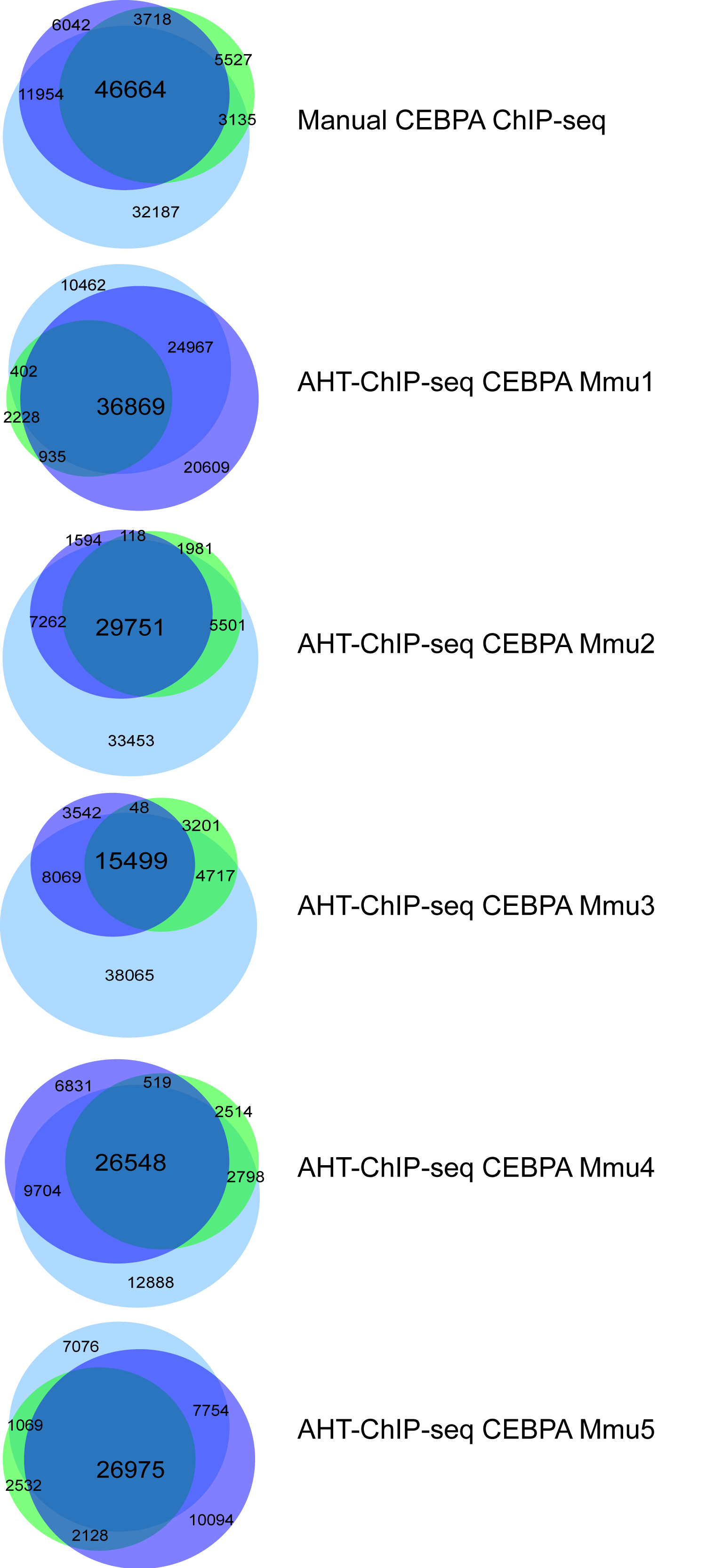

Supplement: Additional file 1 — Supplementary Figures 1-8. [file gb-2013-14-11-r124-S1.zip › Supplementary_figure_4_130520.png]

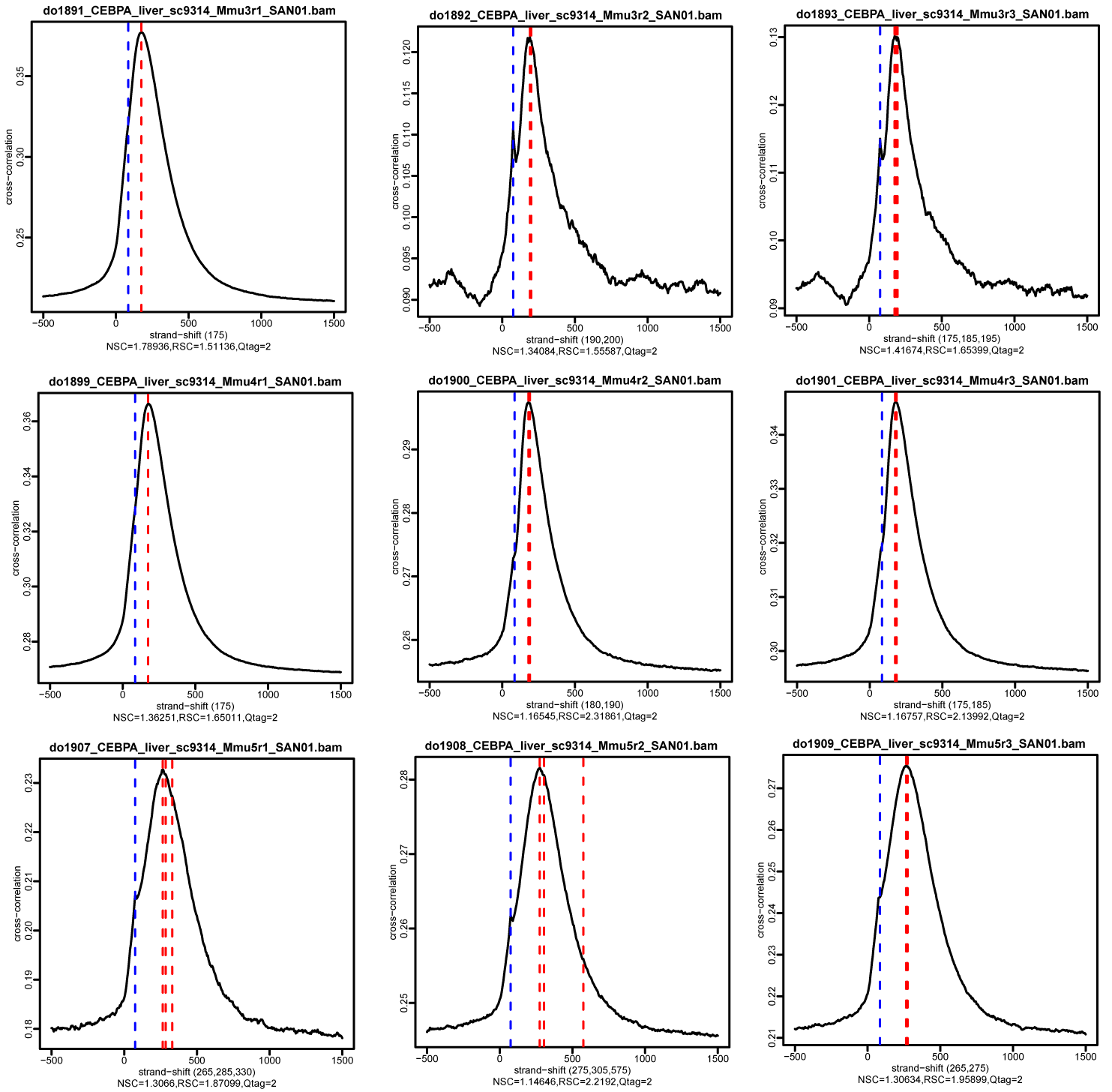

Supplement: Additional file 1 — Supplementary Figures 1-8. [file gb-2013-14-11-r124-S1.zip › Supplementary_figure_3b_130521.png]

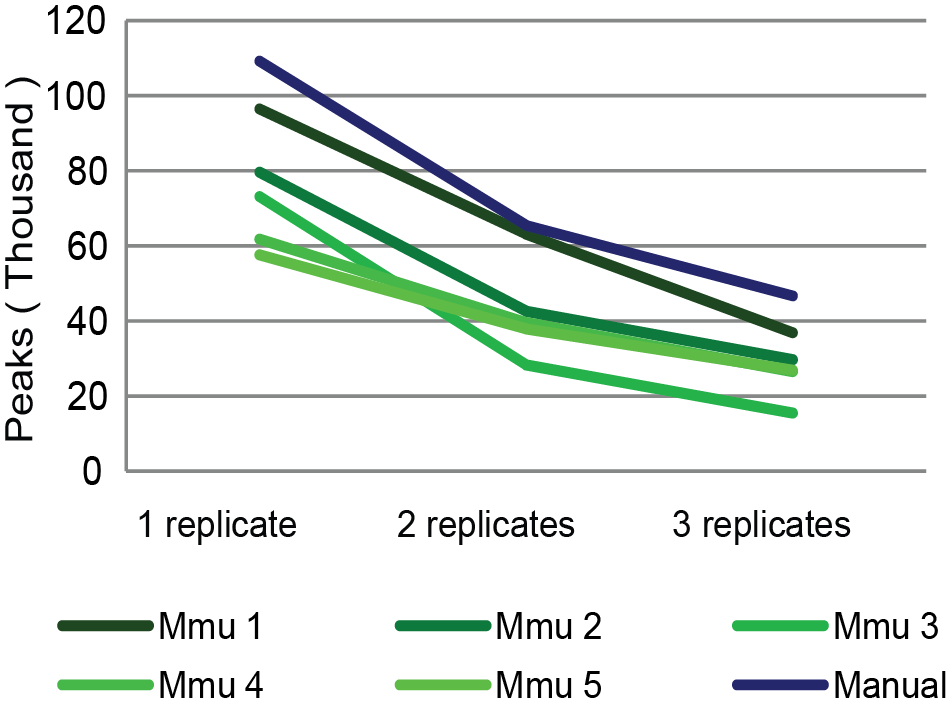

Supplement: Additional file 1 — Supplementary Figures 1-8. [file gb-2013-14-11-r124-S1.zip › Supplementary_figure_5_130521.png]

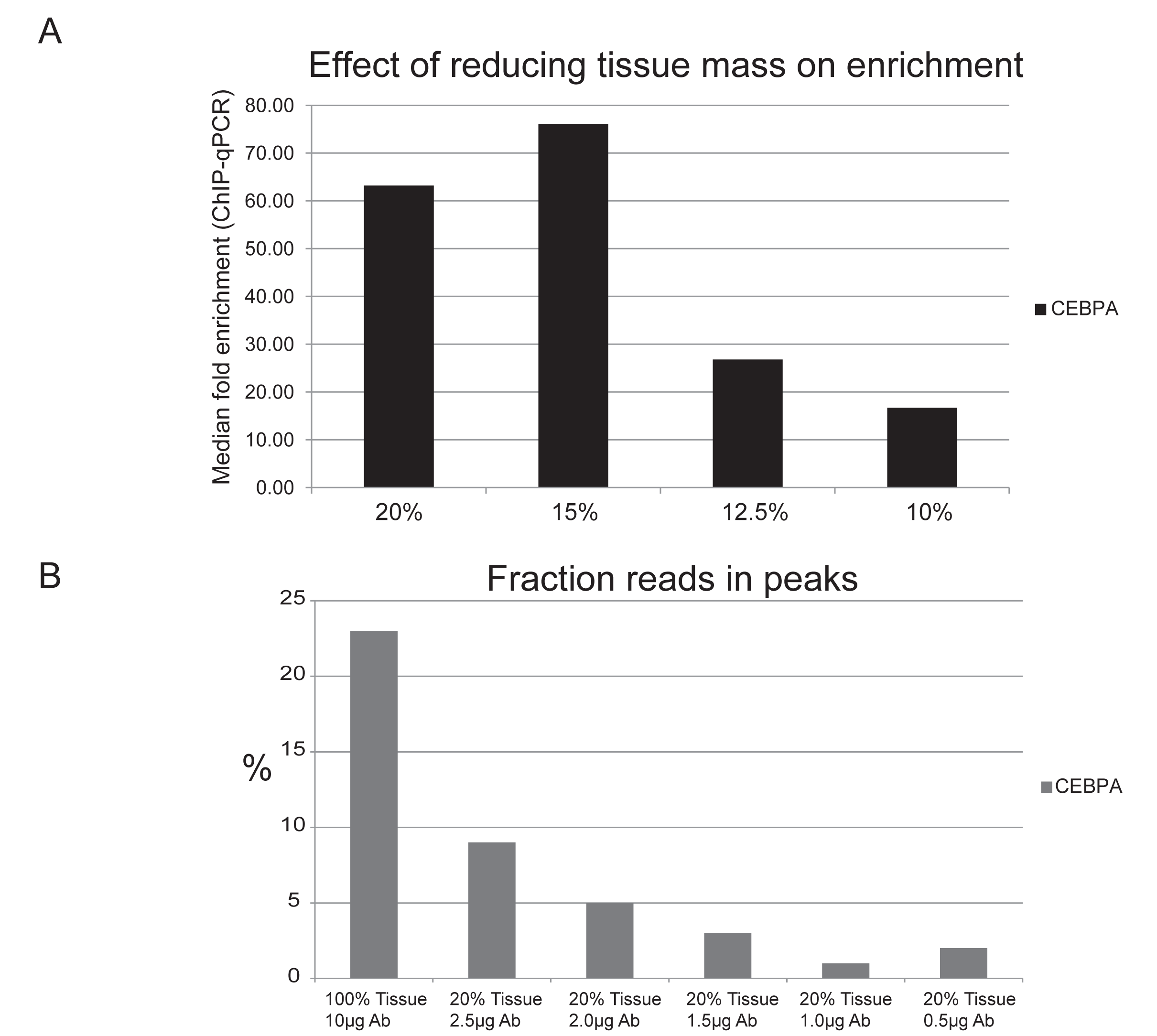

Supplement: Additional file 1 — Supplementary Figures 1-8. [file gb-2013-14-11-r124-S1.zip › Supplementary_figure_1.png]

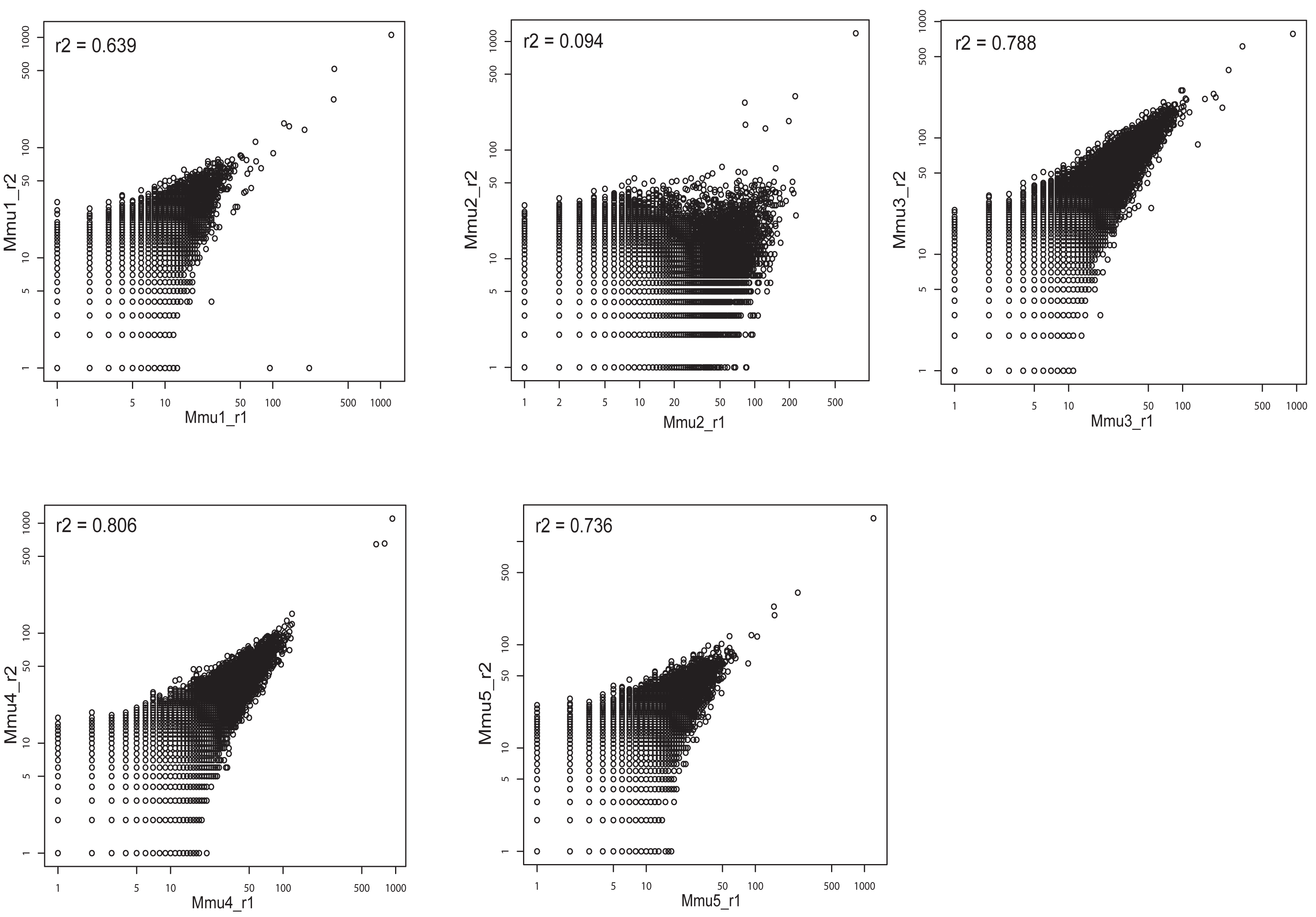

Supplement: Additional file 1 — Supplementary Figures 1-8. [file gb-2013-14-11-r124-S1.zip › Supplementary_figure_6_130521.png]

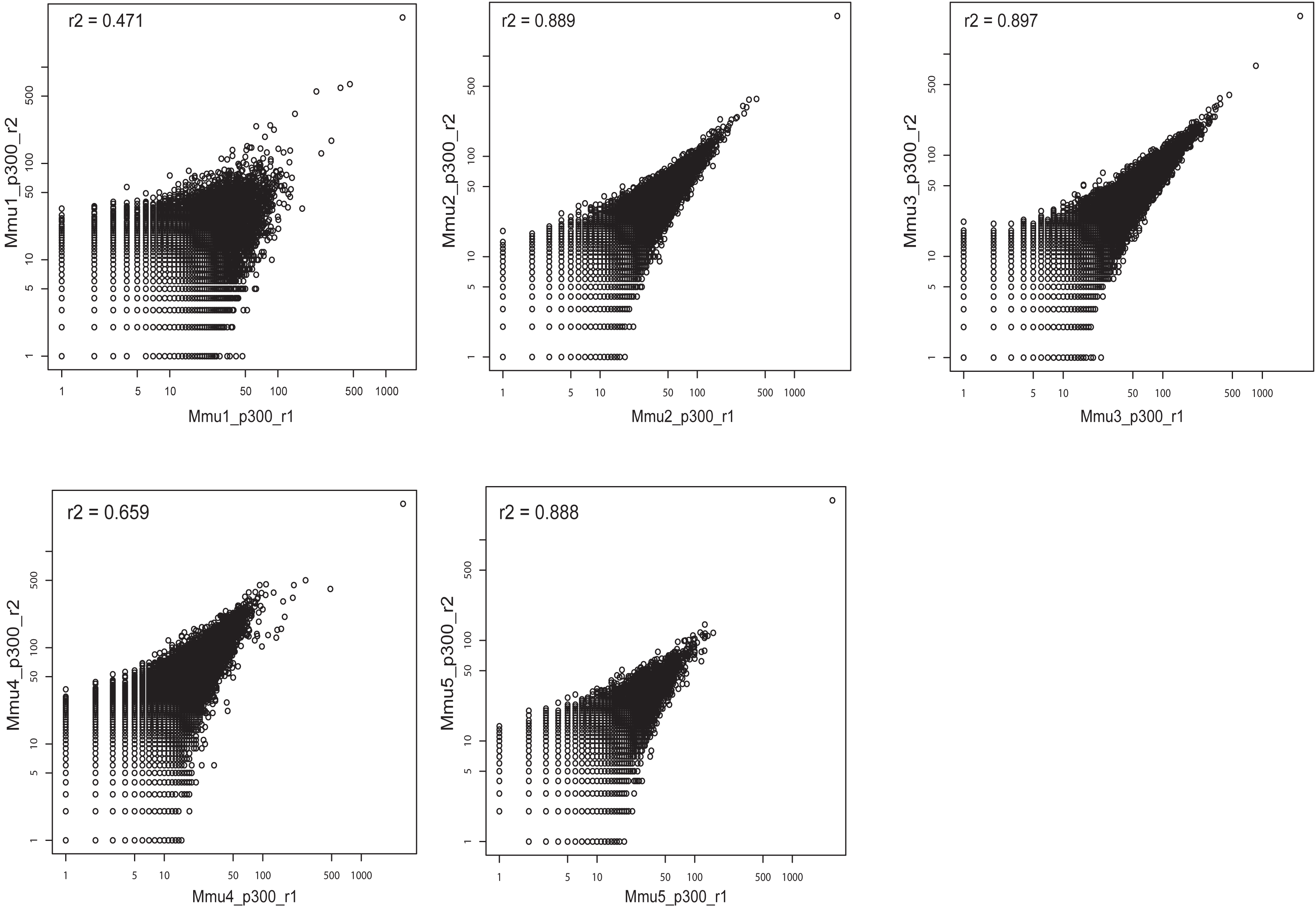

Supplement: Additional file 1 — Supplementary Figures 1-8. [file gb-2013-14-11-r124-S1.zip › Supplementary_figure_7_130521.png]

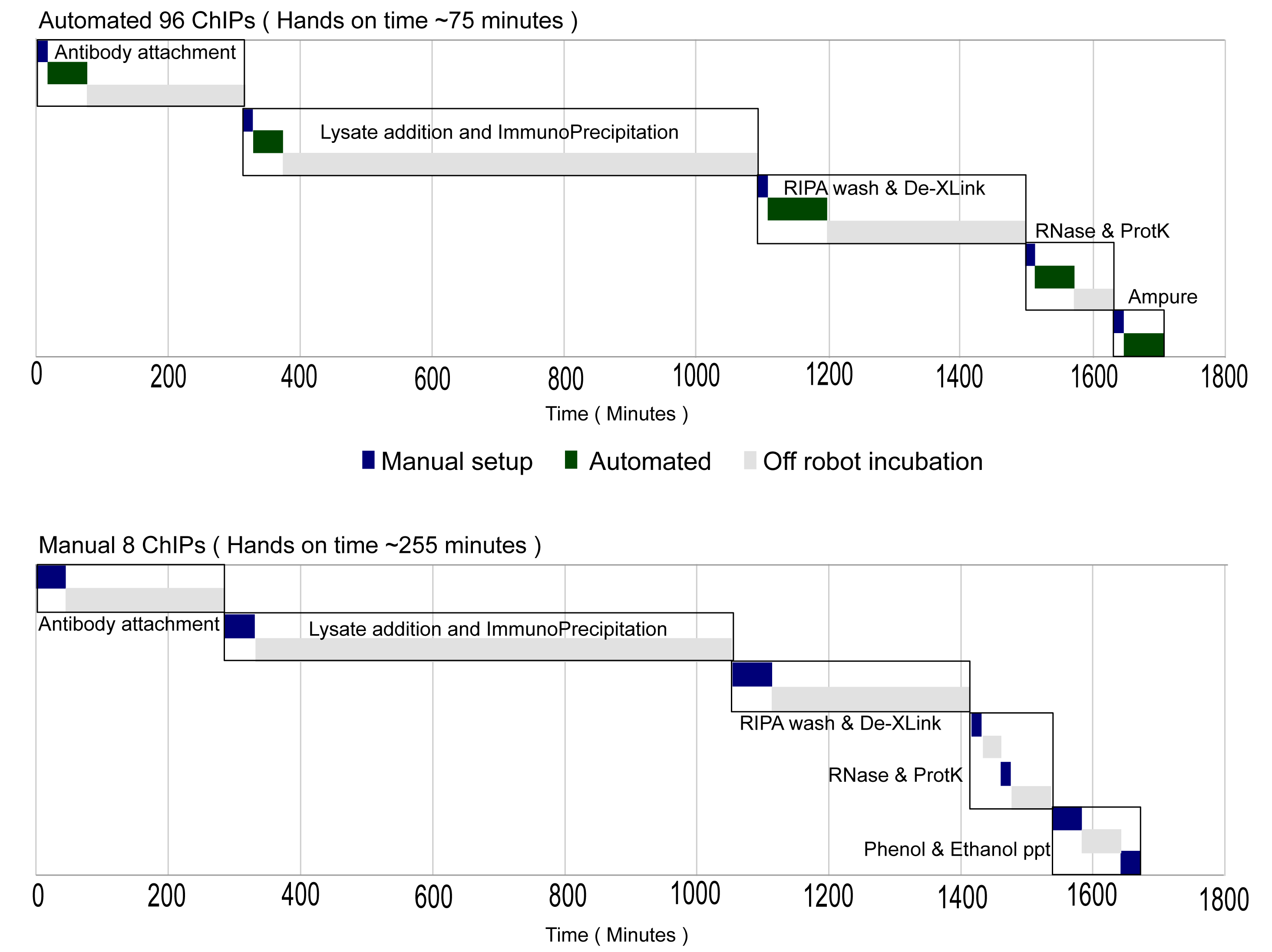

Supplement: Additional file 1 — Supplementary Figures 1-8. [file gb-2013-14-11-r124-S1.zip › Supplementary_figure_2.png]

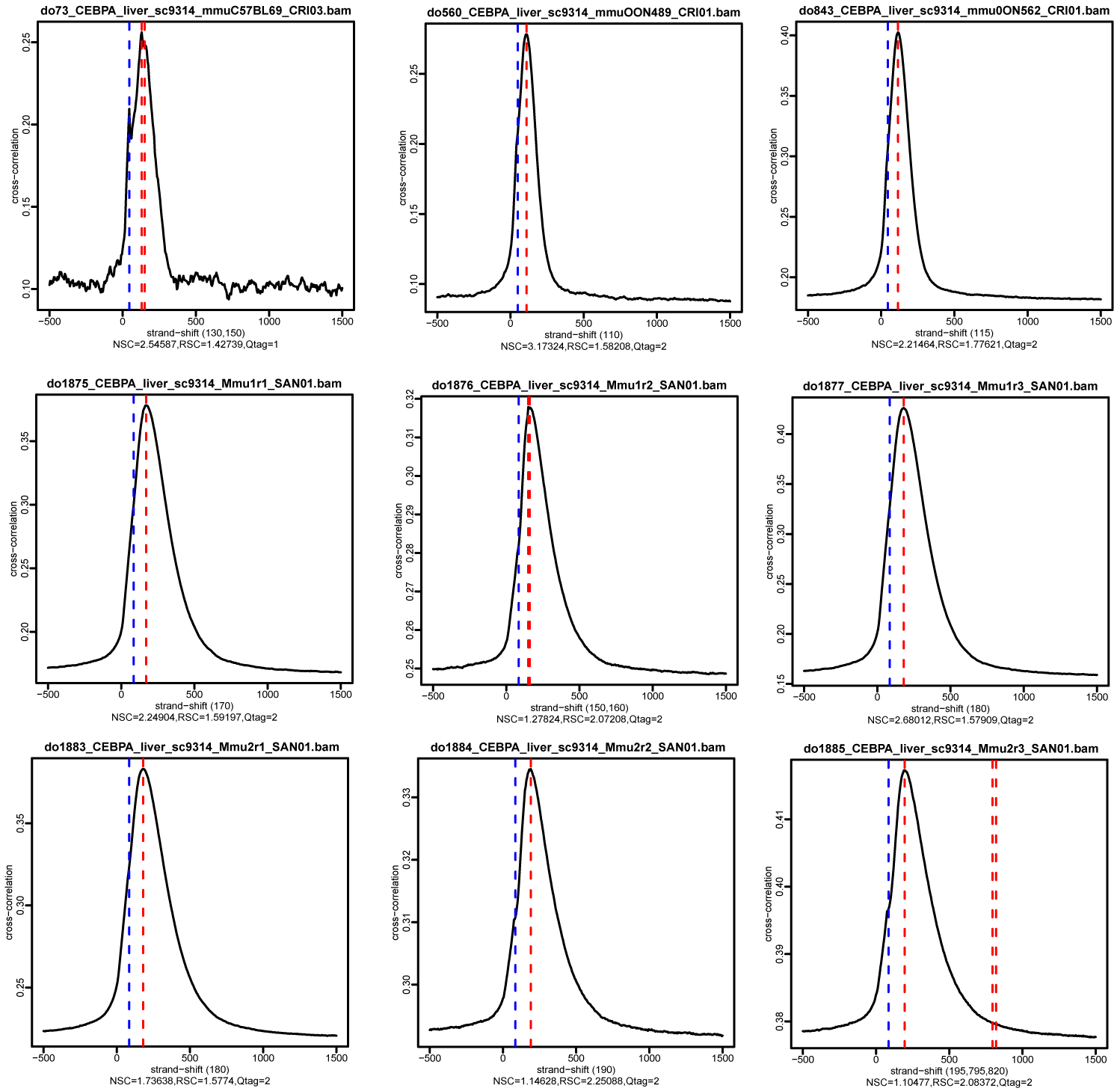

Supplement: Additional file 1 — Supplementary Figures 1-8. [file gb-2013-14-11-r124-S1.zip › Supplementary_figure_3a_130521.png]

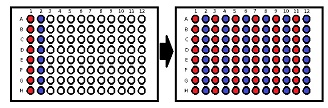

Supplement: Additional file 3 — Agilent file for automated ChIP method. [file gb-2013-14-11-r124-S3.zip › Additional file 3/16 antibodies.jpg]

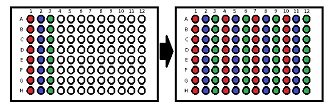

Supplement: Additional file 3 — Agilent file for automated ChIP method. [file gb-2013-14-11-r124-S3.zip › Additional file 3/24 antibodies.jpg]

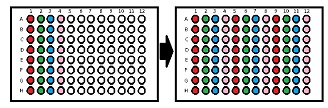

Supplement: Additional file 3 — Agilent file for automated ChIP method. [file gb-2013-14-11-r124-S3.zip › Additional file 3/32 antibodies.jpg]

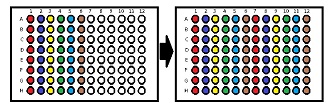

Supplement: Additional file 3 — Agilent file for automated ChIP method. [file gb-2013-14-11-r124-S3.zip › Additional file 3/48 antibodies.jpg]

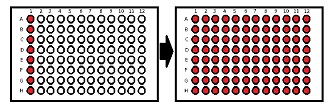

Supplement: Additional file 3 — Agilent file for automated ChIP method. [file gb-2013-14-11-r124-S3.zip › Additional file 3/8 antibodies.jpg]

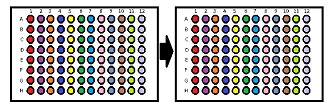

Supplement: Additional file 3 — Agilent file for automated ChIP method. [file gb-2013-14-11-r124-S3.zip › Additional file 3/96 antibodies.jpg]

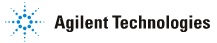

Supplement: Additional file 3 — Agilent file for automated ChIP method. [file gb-2013-14-11-r124-S3.zip › Additional file 3/agilent.jpg]

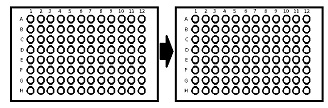

Supplement: Additional file 3 — Agilent file for automated ChIP method. [file gb-2013-14-11-r124-S3.zip › Additional file 3/Blank Plates.jpg]

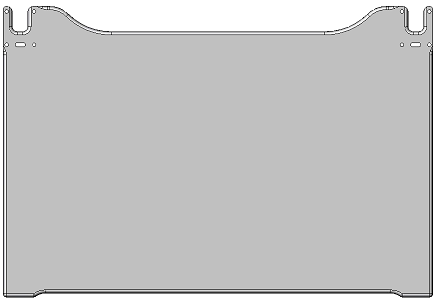

Supplement: Additional file 3 — Agilent file for automated ChIP method. [file gb-2013-14-11-r124-S3.zip › Additional file 3/Bravo Image-small.bmp]

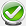

Supplement: Additional file 3 — Agilent file for automated ChIP method. [file gb-2013-14-11-r124-S3.zip › Additional file 3/icon_check_small.png]

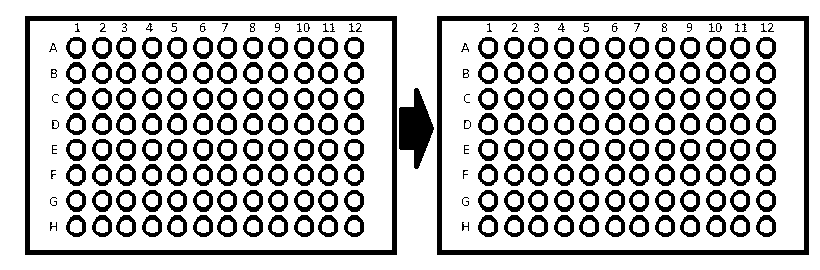

Supplement: Additional file 3 — Agilent file for automated ChIP method. [file gb-2013-14-11-r124-S3.zip › Additional file 3/Master Blank Plates.bmp]

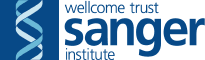

Supplement: Additional file 3 — Agilent file for automated ChIP method. [file gb-2013-14-11-r124-S3.zip › Additional file 3/sanger-logo.png]

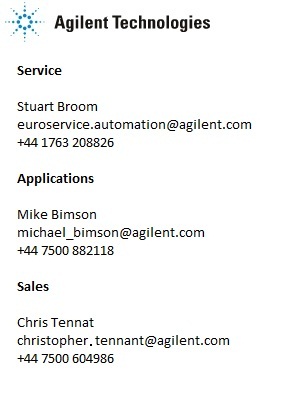

Supplement: Additional file 3 — Agilent file for automated ChIP method. [file gb-2013-14-11-r124-S3.zip › Additional file 3/Support.jpg]
